# Histological and Immunohistochemical Analysis of the Effects of Topical Melatonin Treatment Associated with Collagen Sponge and rhBMP-2 Protein on Bone Remodeling

**DOI:** 10.3390/biom12121738

**Published:** 2022-11-23

**Authors:** Leticia Ferreira Montarele, Dimitrius Leonardo Pitol, Bruno Fiorelini Pereira, Sara Feldman, Valéria Paula Sassoli Fazan, João Paulo Mardegan Issa

**Affiliations:** 1 Faculdade de Odontologia de Ribeirão Preto, Universidade de São Paulo (FORP-USP), Ribeirão Preto 14040-904, Brazil; 2 Department of Biological Sciences, Universidade Federeal de São Paulo—UNIFESP, Diadema 05468-901, Brazil; 3 LABOATEM, Laboratório de Biologia e Engenharia de Tecidos, Faculdade de Medicina, Universidade Nacional de Rosário, Rosário S2002, Argentina; 4 Faculdade de Medicina de Ribeirão Preto, Universidade de São Paulo (FMRP-USP), Ribeirão Preto 14049-900, Brazil

**Keywords:** melatonin, rhBMP-2, bone remodeling, bone repair

## Abstract

Extensive bone defect healing is an important health issue not yet completely resolved. Different alternative treatments have been proposed but, in face of a critical bone defect, it is still very difficult to reach a complete regeneration, with the new-formed bone presenting all morphological and physiological characteristics of a normal, preinjury bone. Topical melatonin use has shown as a promising adjuvant for bone regeneration due to its positive effects on bone metabolism. Thus, to search for new, safe, biological techniques that promote bone repair and favor defect healing, we hypothesized that there is a synergistic effect of melatonin treatment associated with rhBMP-2 to guide bone regeneration. This study aimed to investigate bone repair effects of topical melatonin administration in different concentrations (1, 10, and 100 µg), associated or not with rhBMP-2. Surgical-induced bone defect healing was qualitatively evaluated through histopathological analysis by light microscopy. Additionally, quantitative stereology was performed in immunohistochemistry-prepared tissue to identify angiogenic, osteogenic, and osteoclastogenic factors. Quantification data were compared between groups by the ANOVA/Tukey test and differences were considered significant when *p* < 0.05. Our results showed that the presence of the scaffold in the bone defect hindered the process of bone repair because in the group treated with “blood clot + scaffold” the results of bone formation and immunolabeling were reduced in comparison with all other groups (treated with melatonin alone or in association with rhBMP-2). Statistical analysis revealed a significant difference between the control group (bone defect + blood clot), and groups treated with different concentrations of melatonin in association with rhBMP-2, indicating a positive effect of the association for bone repair. This treatment is promising once it becomes a new safe alternative technique for the clinical treatment of fractures, bone defects, and bone grafts. Our results support the hypothesis of the safe use of the association of melatonin and rhBMP-2 and have established a safe and effective dose for this experimental treatment.

## 1. Introduction

The critical bone defect is characterized by a large bone defect that cannot completely regenerate on its own due to the extent of the injury. Thus, different methods have been developed to establish safe therapeutic approaches, such as the use of synthetic or natural materials that help the bone neoformation process. Among the approaches already well-established in the literature for treating these critical/surgical bone defects are autografts, allografts, and artificial materials that can be used alone or in association with bone grafting. However, these techniques present some disadvantages. Autografts usually cause insufficient bone tissue regeneration, resulting in donor site morbidity. Allografts can cause immunological rejection and inflammation. Fortunately, tissue engineering has been widely explored to overcome these restrictions [1].

Melatonin (N-acetyl-5-methoxytryptamine) is a molecule widely present in nature, occurring in unicellular organisms, plants, fungi, and animals [2]. In most vertebrates, including humans, it is secreted by the pineal gland during the nighttime [3]. Melatonin has been linked to several specific and nonspecific biological processes such as aging, obesity, insulin sensitivity, sexual maturation, antidepressant actions, control of hormone secretions (growth, adrenal, and thyroid hormones), and has antioxidant, oncostatic and osteogenic properties [2,3,4].

Melatonin presents a circadian synchronizer function and can act in all peripheral tissues, including bone tissue [5,6] by keeping metabolism in synchrony with the light–dark cycle. This supports the idea that maximum bone growth may occur during the night when melatonin exhibits its highest plasma level. Bone tissue cells exhibit a rhythm that may be controlled by melatonin [7,8], suggesting that melatonin is an important endogenous biological factor for bone remodeling. Studies have suggested a beneficial effect of melatonin on bone metabolism, including anabolic effects and antiabsorptive effects, which result in osteogenesis. While the antiabsorptive effects are due to the action of melatonin on osteoclasts [9], the anabolic effects on bone remodeling promoted by melatonin are due to its action on osteoblasts. In vitro studies have shown that melatonin promotes proliferation [10] and differentiation [11,12,13] of osteoblast cells. The action of melatonin on osteoblastic cells is a determinant of whether mesenchymal stem cells are targeted for differentiation into osteogenic cells [14]. In vivo studies have shown that the topical application of melatonin can activate osteogenesis around titanium implants, thus favoring osseointegration [15]. Furthermore, melatonin-induced osteoblastic cell formation occurs through increased expression of bone morphogenetic proteins (BMP-2 and BMP-4) involved in osteoblast differentiation [13].

Various carriers, scaffolds, and growth factors such as bone morphogenetic proteins (BMPs) have been tested for bone defect repair [16]. Bone morphogenetic protein type 2, BMP-2, is found in small amounts in mesenchymal cells, collagen fibers, and noncollagenous cells of the bone matrix. BMP-2 is produced by recombinant technology and its osteoinductive role is due to its action on mesenchymal cells, transforming them into osteoprogenitor cells capable of forming bone [17,18,19,20]. However, its isolated topical use is contraindicated due to its rapid dissolution and degradation. Thus, BMP-2 use is recommended in association with biomaterial carriers such as hydroxyapatite, bioactive glass, tricalcium beta phosphate, mineralized and/or demineralized bovine bone, biodegradable polymers, and others [21]. A previous report from our group showed a significant contribution of the rhBMP-2 (5 µg) in critical bone defect repair in rat calvaries [22].

The guided bone regeneration (GBR) technique has shown promising results to enable the repair of large bone defects. Membranes and collagen sponges protect the repair sites from endothelial cells that do not support bone formation, prevent the migration of epithelial cells, and act to prevent initial bone reabsorption [23]. They can also act as a bioactive compartment [24,25]. In addition to these molecular mechanisms, GBR can support the mechanical stability of the bone defect site, favoring the bone remodeling process [26].

Because topically administered melatonin has shown satisfactory results for both dental implantation and injured bone recovery [27,28,29], the synergistic effects of melatonin treatment with a collagen sponge and BMP-2 on bone injury recovery were investigated.

This study aimed to evaluate the bone repair effects of different concentrations of topical melatonin administration, associated or not with rhBMP-2, in surgically induced bone defects, through the guided bone regeneration technique.

## 2. Materials and Methods

A total of 72 adult male Wistar albino rats (250 g) provided by the Central Animal Facility (USP RP—University of São Paulo) were used. These animals were kept in plastic cages (34 × 42 × 37 cm) with a maximum of 3 animals per cage, separated according to the experimental group. The cages were kept in an environment with a controlled temperature (22 ± 2 °C) and a light–dark cycle (12 h light and 12 h dark). The animals were fed with small rodent chow (Nuvital^®^) and water ad libitum throughout the experiment.

For all experimental procedures, the animals were anesthetized with an association of 80 mg/kg of ketamine hydrochloride (Cetamim—Rhobifarma Indústria Farmacêutica Ltd.a, Cotia, SP, Brazil) and 6 mg/kg of xylazine hydrochloride (Zilazin—Rhobifarma Indústria Farmacêutica Ltd.a, Cotia, SP, Brazil), injected in the biceps femoris of the right hind limb. When necessary, anesthesia was supplemented with half the initial applied dose.

Surgical technique: Anesthetized animals were submitted to trichotomy of the skin covering the central region of the calvaria and local disinfection with polyvinylpyrrolidone iodide (Indústria Química e Farmacêutica Rioquímica Ltd., São José do Rio Preto, SP, Brazil). A sagittal incision was made through the skin of the skull cap and periosteum. In the exposed calvaria, bone defects were made bilaterally, both equidistant from the median sagittal suture, using a 6 mm outer diameter drill (Kavo, São Paulo, Brazil), set at 3000 RPM. The calvaria was drilled, under constant and abundant irrigation with 0.9% saline solution.

Treatments and experimental groups: To investigate the isolated and synergistic osteogenic potential of melatonin on the calvaria bone lesion recovery, the animals were distributed into 9 groups, with 8 animals each. Melatonin (1, 10, and 100 µM), bone morphogenetic protein (5 µg BMP-2), and collagen sponge in the form of foam or scaffold (Lumina-Coat Criteria^®,^ São Paulo, SP, Brazil) were applied bilaterally to each animal bone defect. The experimental groups were formed according to the treatment as shown in Table 1.

Postoperative care: After treatment, the edges of the incised skin were repositioned in the midline and sutured using 4.0 silk thread suture (Ethicon, Johnson & Johnson, São José dos Campos, SP, Brazil). Then, each animal received analgesic treatment with a subcutaneous injection of dipyrone (100 mg/kg animal weight), and an intramuscular injection of the Pentabiotic Veterinary Small (Fort Dodge^®^, Campinas, SP, Brazil) antibiotic (0.1 mL/100 g body weight).

Euthanasia and samples processing: After two weeks of the bone defect surgery and treatment, the animals were anesthetized as previously described. After confirmation of the postural reflex and pain sensitivity loss, the animals were decapitated, and the head dissected to obtain the calvaria. Since each animal received the same treatment on both sides of the calvaria, the samples from the left side were used for histological and immunohistochemical analysis for the investigated factors. Samples from the right side were saved for a molecular analysis that will be performed further on.

Histological processing: After decapitation of the animal under anesthesia, the calvaria was removed using appropriate sterile scissors and forceps. The soft tissues present were carefully separated from the bone tissue to obtain the bone fragment containing the defect with a safety margin. These fragments were immersed in the fixative solution (4% buffered paraformoldehyde) for 24 h. Then, the specimens were decalcified in 10% EDTA (neutral pH), changing the solutions once a week (30 to 40 days in total). The specimens were placed for histological processing in the Leica Tp 1020 automated tissue processor and then paraffin-embedded in the Leica central embedding unit. From each sample, semiserial sections of 5 µm thickness were cut, stained by Masson’s trichrome, and observed by light microscopy for bone tissue quantification.

Qualitative analysis by light microscopy: The qualitative analysis of the slices allowed the evaluation of the neoformed bone in the area where the bone defect was created, as well as the differentiation between the existing and the neoformed bone in the experimental groups. For this purpose, a light microscope (AxioImager Z2, Carl Zeiss, Germany) equipped with a digital camera was used. The digital images were analyzed with a 100× magnification objective to evaluate bone tissue by the Masson’s trichrome staining method. The Zeiss software AxioVision was used to evaluate the images.

Quantitative analysis: The quantification of the newly formed bone in each animal was performed using stereological principles, with the software AxioVision (Carl Zeiss, Germany). Using the resources available in the microscope and the software, the entire extent of the bone defect was scanned, and two-dimensional and three-dimensional analyses were performed.

Immunohistochemical analysis: Immunolabeling of proinflammatory cytokine (interleukin 1, 6, and 10, and tnf alpha), angiogenic (CD31 and VEGFR2), osteoclastogenic (OPG), osteogenic (ostecalcin, osteopontin, and RUNX2), and TRAP factors was performed. After blocking the endogenous peroxidase with hydrogen peroxide (10 volumes) for 20 min and the specific bindings with PBS/BSA 2% for one hour each, the sections were immersed in citrate buffer (pH 6.0) solution in a steam chamber for antigen retrieval (Decloaking chamber, by Biocare Medical). After cooling and washing in PBS, the samples were incubated with the primary antibodies for 1 h, using the standard dilution of 1/200 µL. Then, the sections were washed in PBS and incubated for 1 h with the secondary antibody. The abcam immunohistochemistry kit (ab236466 mouse- and rabbit-specific HRP/DAB IHC detection kit micropolymer) was used. The reaction was revealed with diaminobenzidine solution (0.5 mg/mL) in PBS for 1 min. The sections were washed in PBS, counterstained with Harris’ hematoxylin, dehydrated, diaphanized, and covered with coverslips.

Statistical analysis: Quantitative data were submitted to the Shapiro–Wilk normality test, and later to the ANOVA/Tukey test to verify the variance between the groups. Differences were considered significant when *p* < 0.05. The analyses were performed using the GraphPad Prism 5 software.

## 3. Results

The healing period was uneventful in all animals, and no adverse reactions were detected in any of the bone defect sites.

The presence of the scaffold in the bone defect hindered the process of bone repair, because in group 2 (blood clot + scaffold) the results of bone formation and immunolabeling were reduced in comparison with all other groups.

Qualitative and quantitative analysis:

The histological images demonstrate the aspect of bone formation in the repair region, showing a greater amount of bone tissue in groups 6 and 8, which received melatonin 1 and 100 μg, respectively. The presence of bone and blood cells throughout the repair can be observed.

**Figure 1:**
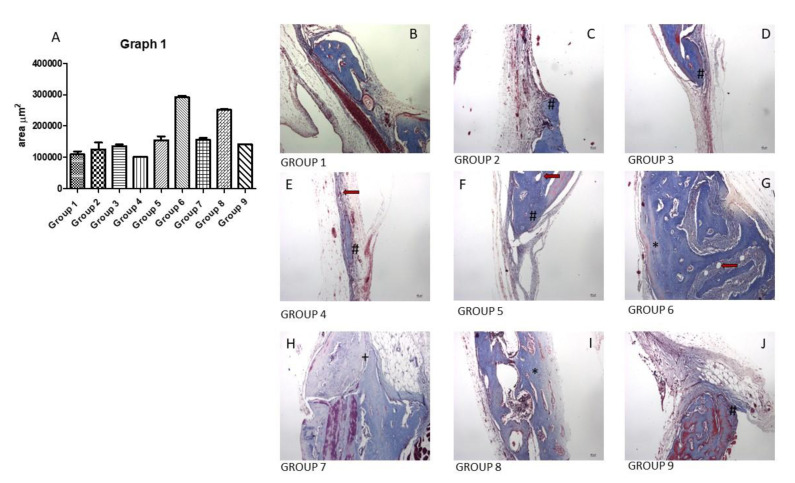
Quantitative and qualitative aspects of the bone defect region in the calvaria of the animals in all experimental groups. (**A**): Graph 1 shows the area of neoformed bone tissue in the bone defect region of the control group and the experimental groups. The graph reveals that there was a significant difference in groups 6 and 8 compared to all other groups. (**B**–**J**): Masson’s trichrome staining was performed to evidence the formation of bone tissue in the repair area. Photomicrographs were taken to show the newly formed bone tissue in the defect area of each group. The presence of osteoblastic cells and newly formed bone tissue can be observed, indicating bone repair of the recipient sites. Masson’s trichrome staining. Scale bar: 46 um. *: greater amounts of neoformed bone tissue, indicating the best results of the therapy used. #: smaller amounts of newly formed bone tissue in the wound region. +: large amounts of newly formed bone tissue, but without significant difference. Red arrow: bone gaps.

Figure 1 shows a graph with the neoformed bone tissue area and histological images of the bone recovery area of each experimental group. Bone tissue and blood cells can be seen alongside the neoformed bone tissue in the defect area. The neoformed bone tissue area was significantly larger in groups 6 and 8.

Statistical analysis revealed a significant difference (*p* < 0.05) between the control group (group 1), and experimental groups 5, 6, 7, and 8 (groups with different concentrations of melatonin in association with rhBMP-2), indicating a positive effect of the association for bone repair. Other differences observed were: group 2 vs. groups 6 and 8; group 3 vs. groups 6 and 8; group 4 vs. groups 5, 6, 7, and 8; group 5 vs. groups 6 and 8; group 6 vs. groups 7 and 9; group 7 vs. group 8; and group 8 vs. group 9

Immunohistochemistry Analysis:

The results obtained by immunohistochemistry confirmed the light microscopy observations that the presence of the scaffold in the bone lesion impairs the action of the substances tested for bone regeneration improvement, as, in group 2 (clot + scaffold), antibody labeling was very low.

**Figure 2:**
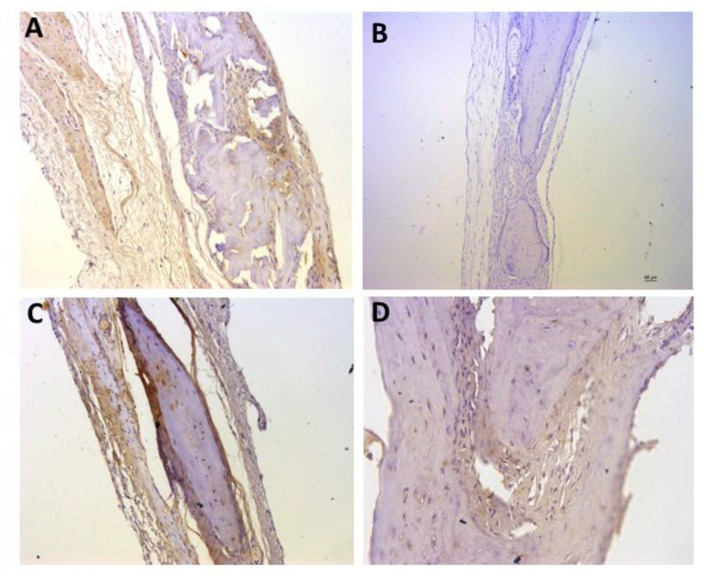
Representative immunohistochemistry images for VEGF staining. (**A**) control group; (**B**) negative control; (**C**) group 2 with marking considered (++); (**D**) group 5 with marking considered (++), the most observed staining pattern among the groups analyzed. Harris’ hematoxylin counterstained. Scale bar: 46 µm.

**Figure 3:**
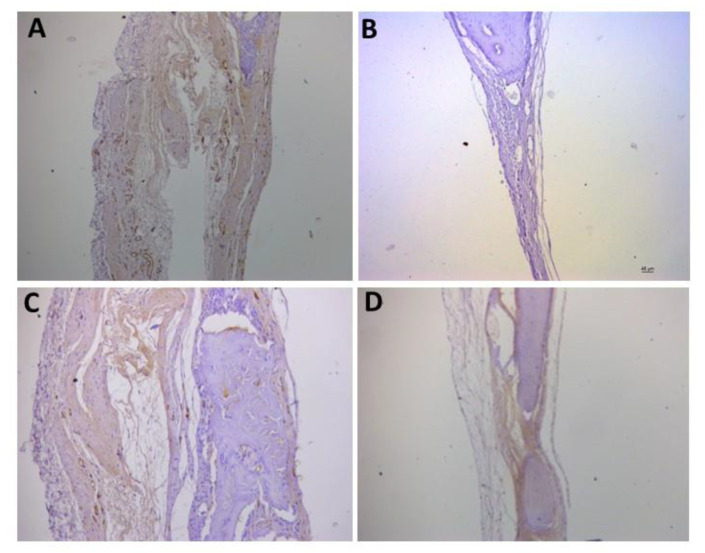
Representative immunohistochemistry images for CD31 staining. (**A**) control group; (**B**) negative control; (**C**) group 2 with marking considered (+); (**D**) group 6 with marking considered (+); the most observed staining pattern among the groups analyzed. Harris’ hematoxylin counterstained. Scale bar: 46 µm.

**Figure 4:**
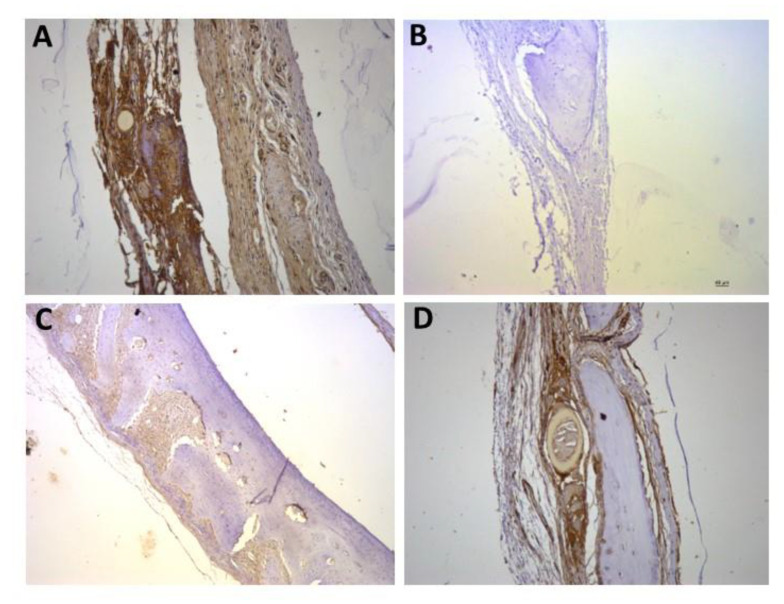
Representative immunohistochemistry images for OPG staining. (**A**) control group; (**B**) negative control; (**C**) group 2 with marking considered (+); (**D**) group 6 with marking considered (++++); the most observed staining pattern among the groups analyzed. Harris’ hematoxylin counterstained. Scale bar: 46 µm.

**Figure 5:**
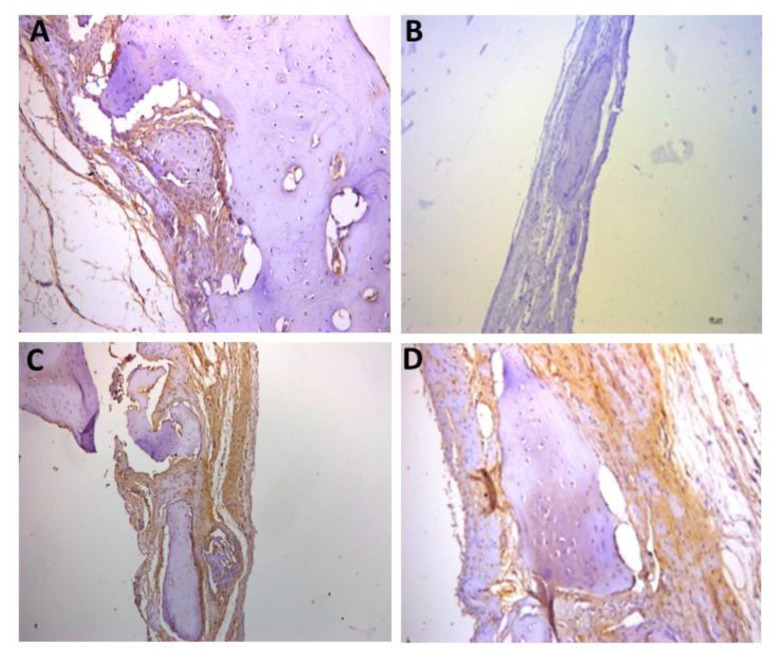
Representative immunohistochemistry images for IL1β staining. (**A**) control group; (**B**) negative control; (**C**) group 2 with marking considered (++); (**D**) group 7 with marking considered (++); the most observed staining pattern among the groups analyzed. Harris’ hematoxylin counterstained. Scale bar: 46 µm.

**Figure 6:**
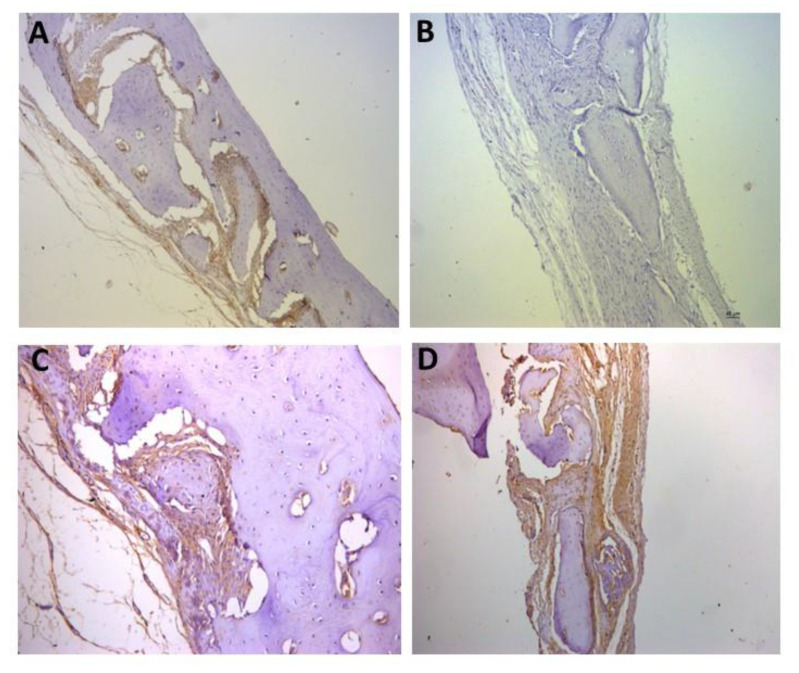
Representative immunohistochemistry images for IL6 staining. (**A**) control group; (**B**) negative control; (**C**) group 2 with marking considered (+++); (**D**) group 3 with marking considered (++++); the most observed staining pattern among the groups analyzed. Harris’ hematoxylin counterstained. Scale bar: 46 µm.

**Figure 7:**
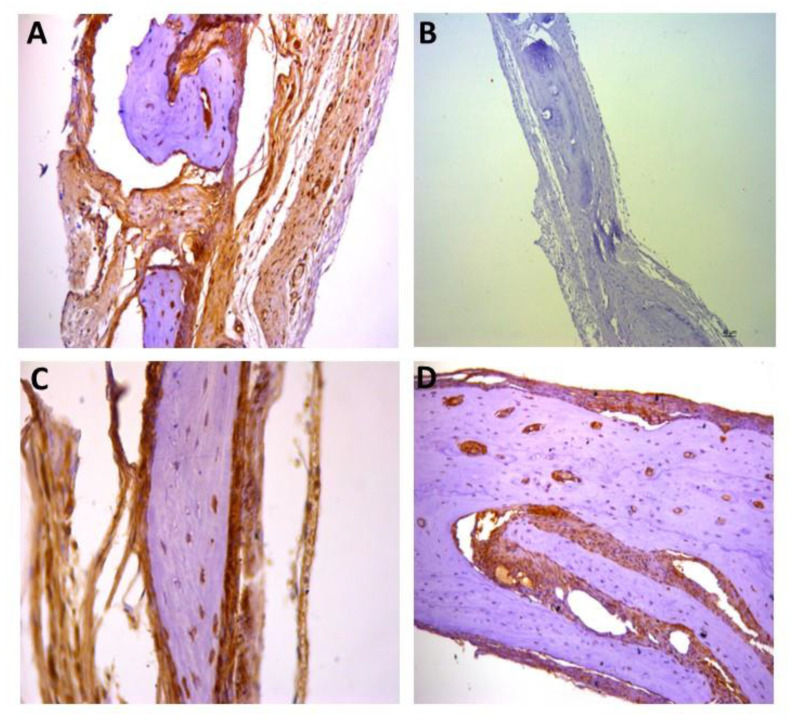
Representative immunohistochemistry images for IL10 staining. (**A**) control group; (**B**) negative control; (**C**) group 2 with marking considered (+++); (**D**) group 5 with marking considered (+++); the most observed staining pattern among the groups analyzed. Harris’ hematoxylin counterstained. Scale bar: 46 µm.

**Figure 8:**
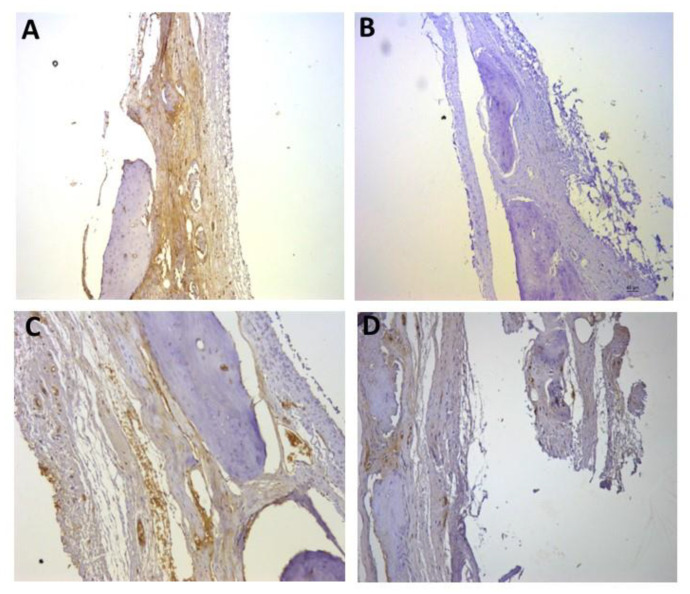
Representative immunohistochemistry images for osteocalcin staining. (**A**) control group; (**B**) negative control; (**C**) group 2 with marking considered (+); (**D**) group 8 with marking considered (+++); the most observed staining pattern among the groups analyzed. Harris’ hematoxylin counterstained. Scale bar: 46 µm.

**Figure 9:**
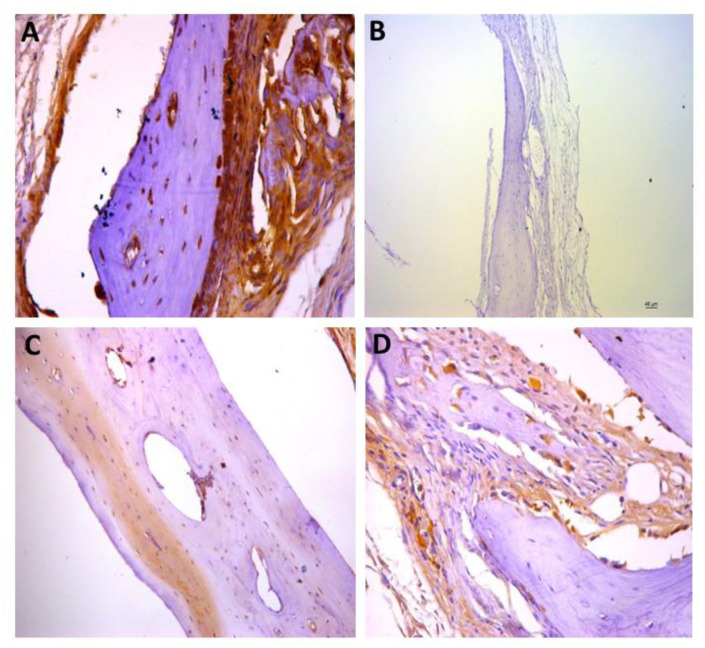
Representative immunohistochemistry images for osteopontin staining. (**A**) control group; (**B**) negative control; (**C**) group 2 with marking considered (++); (**D**) group 9 with marking considered (++++); the most observed staining pattern among the groups analyzed. Harris’ hematoxylin counterstained. Scale bar: 46 µm.

**Figure 10:**
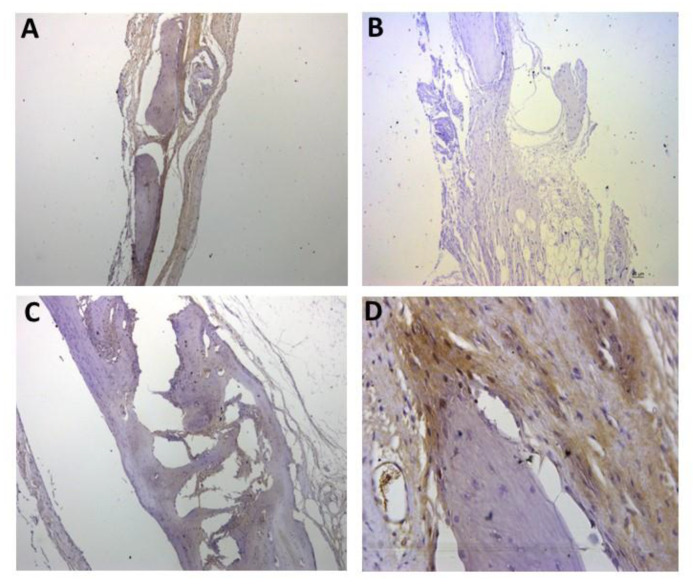
Representative immunohistochemistry images for RUNX2 staining. (**A**) control group; (**B**) negative control; (**C**) group 2 with marking considered (+); (**D**) group 3 with marking considered (++); the most observed staining pattern among the groups analyzed. Harris’ hematoxylin counterstained. Scale bar: 46 µm.

**Figure 11:**
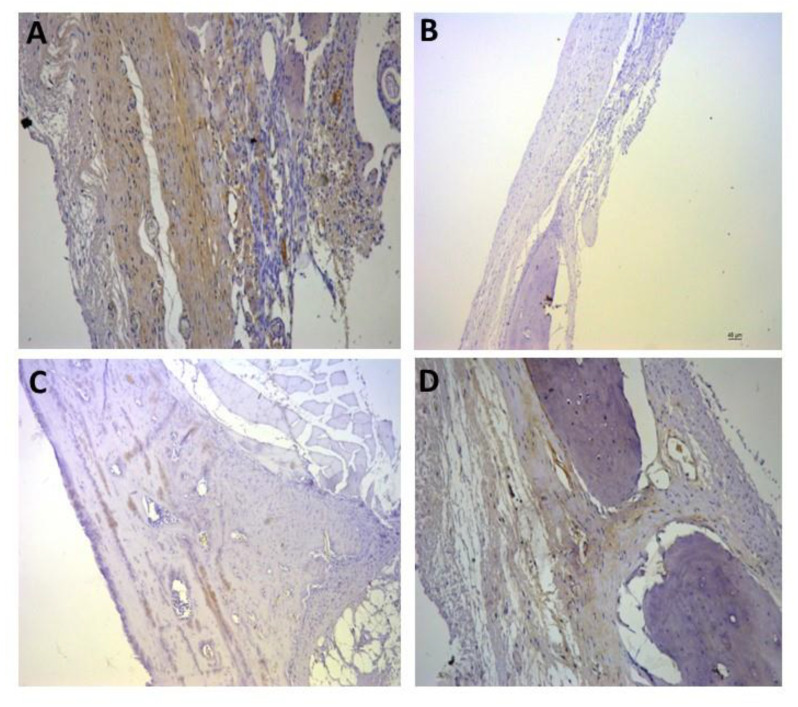
Representative immunohistochemistry images for TRAP staining. (**A**) control group; (**B**) negative control; (**C**) group 2 with marking considered (+); (**D**) group 4 with marking considered (++); the most observed staining pattern among the groups analyzed. Harris’ hematoxylin counterstained. Scale bar: 46 µm.

For the representative immunohistochemistry images (Figure 2, Figure 3, Figure 4, Figure 5, Figure 6, Figure 7, Figure 8, Figure 9, Figure 10 and Figure 11), A shows the control group, B the negative control, C group 2, and D a group representing the most frequent reaction among all groups. Group 2 was chosen to be shown in all images because its quantitative staining stood out for the low reaction for all investigated factors, indicating an inhibitory effect of the scaffold.

The images of the immunohistochemistry of angiogenic factors (VEGF and CD31) are shown in Figure 2 and Figure 3, respectively. It can be observed in Table 2 and in the images that the positive reaction of these factors was generally low. Figure 2C shows the group 2 (++) staining, and D shows group 5 (++) staining, which was the most observed staining pattern among all groups. Figure 3C shows group 2 (+) staining, and D shows group 6 (+) staining, the most observed staining pattern among all groups.

The immunolabeling for osteoprogesterin (OPG), which is a factor for osteoclastogenesis, is shown in Figure 4. In C, the labeling in group 2 is represented as (+) and in D, the most observed staining pattern among the groups (++++) is represented by group 6.

Figure 5, Figure 6 and Figure 7 show the positive reaction of interleukins 1β, 6, and 10, respectively. In all of them, group 2 also showed very weak labeling. For interleukin 1β, the marking (++) was the most predominant in all groups (Figure 5D). For interleukin 6, the marking (+++) was the most common (Figure 6D), as well as for interleukin 10, for which the marking (+++) also prevailed in all groups (Figure 7D).

Figure 8, Figure 9 and Figure 10 show the immunostaining for osteogenic factors: osteopontin, osteocalcin, and RUNX2, respectively. In all of them the lowest labeling also occurred in group 2. Figure 9D shows significant labeling (++++) of osteopontin, which is a strong marker of bone formation, justified by the effect of rhBMP-2.

Figure 11 represents the immunostaining for TRAP. Once again, the lowest marking (+) occurred in group 2 (Figure 11C), and the most observed staining pattern among the other groups (++), is represented by group 4 (Figure 11D).

## 4. Discussion

The present study investigated the topical use of melatonin in different concentrations, associated or not with rhBMP-2, to improve bone repair in surgical bone defects using the GBR technique. This treatment is promising once it becomes a new, safe alternative technique for the clinical treatment of fractures, bone defects, and bone grafts. Our results proved the safe use of the proposed association and established a safe and effective dose for this experimental treatment. There are no literature reports that have evaluated bone repair using the methodologies described in the present study.

The high-power resolution histological analyses used in this study allowed a better comprehension of how the bone tissue reacted to the different treatments as well as to evaluate the biological stages of bone repair. This is because most studies use single or multiple nonserial tissue sections, reducing the sensitivity of the histological data. The use of stereology expands the range of investigated variables and provides greater accuracy in tissue reaction quantification, improving the quantitative analysis methods [30].

Rat calvaria defects are widely used for histological and imaging analysis due to the stability of the bones in terms of movements. The experimental model used in this study was designed to reproduce a critical bone defect since these are the most complex defects for regeneration, due to the difficulty of spontaneous complete healing. Critical bone defects in the calvaria are used to evaluate different materials as viable and safe alternatives for restoring bone architecture, mainly in the craniofacial region [31,32].

To choose a material or substance that favors bone regeneration, the material’s safety to the organism is fundamental. So, it is necessary to take into consideration some main properties of the chosen material, such as biocompatibility, osteogenesis, osteoinduction, and osteoconduction [33,34].

The autologous blood clot presents the capacity by itself to be a physiological agent to induce bone formation due to the presence of several cells that favor this process. Grgurevic L., et al. (2020) demonstrated that the clot can act as a native rhBMP transporter for bone formation [35].

There are reports of melatonin, GBR, and rhBMP-2 used in association to provide effective bone repair. The authors stated that melatonin exerts an osteogenic effect, while rhBMP-2 provides osteoinduction, and the collagen sponge provides osteoconduction. Currently, melatonin is known to have properties related to bone metabolism, and its use favors bone repair due to osteogenic and antiosteoclastic properties [9,36,37,38].

Wang et al. (1990) demonstrated that the dose of rhBMP-2 is critical for bone formation [39]. In our study, the choice of a dose of 5 μg of rhBMP-2 was based on studies from Kotake et al. (2015) and Gonzaga et al. (2019), that used 5 μg of this protein and showed a significant contribution of it for bone formation in critical defects [22,32].

rhBMP-2 is a widely studied morphogenetic protein with an important role in biological processes, mainly related to bone tissue. It presents an osteoinductive role capable of repairing and regenerating bone tissue as it acts by sending morphogenic signals for migration, proliferation, and differentiation of mesenchymal cells into bone tissue. However, its use alone has the disadvantage of being of rapid dissolution in the medium in which it is applied, requiring a carrier [40,41].

The technique of guided bone regeneration (GBR) has become a well-documented and successful clinical procedure that has been developed to help the reconstruction of alveolar bone. The fundamental principle of GBR is to function as a barrier, preventing nonosteogenic tissues, such as epithelial and conjunctival cells, from invading the bone repair area. Moreover, it also creates a bioactive compartment with the ability to form a structure that ensures osteoconduction [42] Thus, the use of GBR is already well-established in the literature for therapies aimed at bone repair [43,44]

Jung et al. (2003) concluded that the combination of a xenogenic bone mineral substitute with rhBMP-2 can enhance the maturation process of bone regeneration, demonstrating the potential of rhBMP-2 to predictably improve and accelerate guided bone regeneration therapy [45]. Schorn et al. (2017) evaluated the association of rhBMP-2 and VEGF with a collagen carrier and also had good results with vertical bone formation [46]. Wikesjö et al. (2004) also obtained good results with the use of rhBMP-2 associated with bone regeneration [47,48,49,50,51].

As already mentioned, osteoinductive proteins require a carrier due to their short biological half-life, and for them to be released in a gradual and prolonged manner. They also act as a delivery system and as a support for cell growth. The combination of an osteoinductive protein (rhBMP-2) with an osteoconductive material makes it possible to overcome some of the difficulties encountered with current bone regeneration techniques, justifying the choice of GBR in this study.

In addition to acting on mesenchymal cell differentiation, osteoblast regulation, chemotaxis, and mitosis during bone repair [52], the use of rhBMP-2 effectively accelerates bone regeneration.

Given all this, a great bone repair was predicted in groups 3, 4, 5, and 9, as they presented the isolated associations between melatonin and scaffold, and rhBMP-2 and scaffold. In group 9, in which the association was blood clot + scaffold + 5 μg rhBMP-2, there was no great bone formation response, as observed in groups 3, 4, and 5 (clot + scaffold + melatonin in different concentrations, 1, 10, and 100 μg, respectively). It was expected that in these groups high bone formation could be observed due to the osteogenic effects of the isolated use of melatonin or rhBMP-2 since they are agents that enhance bone formation in the repair area. This result is in agreement with the findings obtained by Sampath et al. (1981) which revealed that BMP associated with collagen support is capable of inducing bone formation in different sites, favoring bone regeneration [53]. Likewise, Burkus et al. (2009) demonstrated that the use of rhBMP-2, applied within an absorbable collagen sponge, was able to induce bone formation [54]. From our results, we can infer that the presence of the scaffold inhibited bone regeneration at the defect site, maybe acting as a physical barrier for the melatonin and rhBMP-2 migration and action. Groups 6 (blood clot + scaffold + 1 μg of melatonin + 5 μg of rhBMP-2) and 8 (blood clot + scaffold + 100 μg of melatonin + 5 μg of rhBMP-2) presented the same substance combination, with a difference in the concentration of melatonin, and were the only ones that showed significantly increased bone formation compared to the other groups. This significant difference can be explained by the action of rhBMP-2, as it is, by itself, an inducer of bone formation [55,56,57].

It was observed in groups 3, 4, and 5 that melatonin had practically no action on bone regeneration, once again indicating that it was highly impaired by the scaffold in our experimental model, differing from studies that revealed bone formation with the use of melatonin alone [58,59].

The qualitative analysis revealed that experimental groups 6, 7, 8, and 9 visually presented a greater amount of reparative bone trabeculae in the area of the bone defect. However, there was still a large area to be repaired when compared to the other groups.

The histomorphometric analysis revealed the best result for group 6 (blood clot + scaffold + 1 μg of melatonin + 5 μg of rhBMP-2). The lowest dose of melatonin used showed a higher osteogenic response when compared to the other groups.

Immunohistochemistry was performed using biomarkers of angiogenic (cd31 and VEGF, osteoclastogenic (OPG), osteogenic (osteocalcin, osteopontin, and RUNX2), proinflammatory (interleukin 1, 6, and 10, and TNF alpha), and TRAP. In the results obtained by immunohistochemistry, it is possible to note the weak labeling of factors involved in bone repair (angiogenic and osteogenic factors), especially in group 2. This reinforces the hypothesis of inhibition of bone repair caused by the use of the scaffold. Despite this, once again, the best results were obtained in groups 6 and 8, reinforcing the histologic observations of a better regeneration process in groups treated with melatonin 1 and 100 µg, associated with rhBMP-2, respectively.

## 5. Conclusions

According to the methodologies used and the results obtained in this study, the association of melatonin (1 µg) with 5 μg rhBMP-2, through the technique of guided bone regeneration, was able to increase the bone repair of critical defects in rat calvaria, but this process was also inhibited by the presence of the scaffold, in all groups.

## Figures and Tables

**Table 1:** Distribution of treatments and experimental groups.

Groups	Treatments
Group 1	blood clot
Group 2	blood clot + Scaffold
Group 3	blood clot + Scaffold *+* Melatonin 1 µg
Group 4	blood clot + Scaffold *+* Melatonin 10 µg
Group 5	blood clot + Scaffold *+* Melatonin 100 µg
Group 6	blood clot + Scaffold *+* Melatonin 1 µg + rhBMP-2
Group 7	blood clot + Scaffold *+* Melatonin 10 µg + rhBMP-2
Group 8	blood clot + Scaffold *+* Melatonin 100 µg + rhBMP-2
Group 9	blood clot *+* Scaffold + rhBMP-2

**Table 2:** Immunohistochemistry reaction intensity for all investigated factors in all experimental groups.

	Group 1	Group 2	Group 3	Group 4	Group 5	Group 6	Group 7	Group 8	Group 9
OPG	++++	+	+++	+++	+++	++++	++	++++	+++
CD31	+	+	+	+	+	+	+	+	+
IL1β	++	++	++	+++	++	++	++	++	++
IL6	+++	+++	+++	+++	+++	+++	+++	+++	+++
IL10	+++	+++	+++	+++	+++	+++	+++	+++	+++
Osteocalcin	+++	+	++	++	++	+++	+++	+++	+++
Osteoprogeterin	++++	++	++	++	++	++++	+++	++++	++++
RUNX2	++	+	++	++	++	+++	++	+++	+++
TRAP	++	+	++	++	++	++	++	++	++
VEGF	++	++	++	++	++	++	+++	++	++

## Data Availability

Not applicable.
